# Functionality of Cricket and Mealworm Hydrolysates Generated after Pretreatment of Meals with High Hydrostatic Pressures

**DOI:** 10.3390/molecules25225366

**Published:** 2020-11-17

**Authors:** Alexandra Dion-Poulin, Myriam Laroche, Alain Doyen, Sylvie L. Turgeon

**Affiliations:** Department of Food Sciences, Institute of Nutrition and Functional Foods (INAF), Université Laval, Quebec City, QC G1V 0A6, Canada; alexandra.dion-poulin.1@ulaval.ca (A.D.-P.); myriam.laroche.6@ulaval.ca (M.L.); alain.doyen@fsaa.ulaval.ca (A.D.)

**Keywords:** entomophagy, *Gryllodes sigillatus*, *Tenebrio molitor*, edible insect meals, protein hydrolysate, high hydrostatic pressures, functional properties

## Abstract

The low consumer acceptance to entomophagy in Western society remains the strongest barrier of this practice, despite these numerous advantages. More positively, it was demonstrated that the attractiveness of edible insects can be enhanced by the use of insect ingredients. Currently, insect ingredients are mainly used as filler agents due to their poor functional properties. Nevertheless, new research on insect ingredient functionalities is emerging to overcome these issues. Recently, high hydrostatic pressure processing has been used to improve the functional properties of proteins. The study described here evaluates the functional properties of two commercial insect meals (*Gryllodes sigillatus* and *Tenebrio molitor*) and their respective hydrolysates generated by Alcalase^®^, conventionally and after pressurization pretreatment of the insect meals. Regardless of the insect species and treatments, water binding capacity, foaming and gelation properties did not improve after enzymatic hydrolysis. The low emulsion properties after enzymatic hydrolysis were due to rapid instability of emulsion. The pretreatment of mealworm meal with pressurization probably induced protein denaturation and aggregation phenomena which lowered the degree of hydrolysis. As expected, enzymatic digestion (with and without pressurization) increased the solubility, reaching values close to 100%. The pretreatment of mealworm meal with pressure further improved its solubility compared to control hydrolysate, while pressurization pretreatment decreased the solubility of cricket meal. These results may be related to the impact of pressurization on protein structure and therefore to the generation of different peptide compositions and profiles. The oil binding capacity also improved after enzymatic hydrolysis, but further for pressure-treated mealworm hydrolysate. Despite the moderate effect of pretreatment by high hydrostatic pressures, insect protein hydrolysates demonstrated interesting functional properties which could potentially facilitate their use in the food industry.

## 1. Introduction

Recently, the interest in entomophagy, defined as the practice of eating insects, is growing due to its environmental and nutritional benefits compared to other livestock [1,2,3,4,5,6,7,8,9]. Edible insects have been targeted as potential alternative protein sources to resolve the problem of a global food crisis since, overall, their protein content is over 50% [4,10]. Despite the nutritional and sustainable advantages of insect consumption, low acceptability and negative consumer perception (insects as pests, disgusting and unsafe) of this unconventional food matrix remains the main issue in Western societies for the development of this food sector [11]. However, several studies have shown that acceptability is improved when incorporation of insects in food is unrecognizable [12,13,14,15,16]. Consequently, the use of insect ingredients, such as insect meals, concentrate or isolate, may be a promising strategy to improve consumer acceptance [11,15].

Insect meal is obtained simply by grinding whole dried insects. Oven-drying is the most widely used method for production of edible insect meal at a commercial scale [17,18]. However, studying other conventional and emerging drying technologies demonstrated that processing parameters largely influence the protein functionality [18]. More specifically, Kröncke et al. studied the effect of different drying methods on the solubility of mealworm (*Tenebrio molitor*) proteins [19]. Solubility is a very important functional property because it influences other functional properties such as emulsifying properties [3,20]. Thus, the solubility of mealworm (*T. molitor*) proteins decreased significantly, from 53% in the fresh state to only 14% after oven-drying [19]. This decrease in solubility was caused mainly by protein denaturation during heat treatment, which unfolds and exposes previously hidden hydrophobic groups [21].

Enzymatic hydrolysis is widely used to improve and modify protein functionality from a wide range of protein sources [22,23]. Currently, few studies are available on the functional properties of insect hydrolysates generated after enzymatic digestion. As examples, Wang et al. showed that the solubility of housefly (*Musca domestica*) protein was greater than 90% after enzymatic hydrolysis, and Hall et al. showed that enzymatic hydrolysis not only improved the solubility of cricket (*Gryllodes sigillatus*) proteins but also their emulsifying and foaming properties, which depend on hydrolysis parameters [3,24]. Purschke et al. improved the protein solubility, foaming properties and oil binding capacity of a migratory cricket (*Locusta migratoria* L.) through enzymatic hydrolysis using several proteases (alone or in combination), enzyme/substrate ratios and hydrolysis times [20]. Several protein pretreatment methods, such as microwaves [25,26], ultrasound [26,27,28] and high-voltage pulsed electric field [29,30,31], have been shown to improve hydrolysis rate and enhance bioactive peptide production. However, recently, interest in the use of high hydrostatic pressure for protein pretreatment is growing [32].

High hydrostatic pressures influence protein functionality by modulating the structures and conformation of the protein [33,34]. The denaturation of proteins caused by disruption of non-covalent bonds (hydrogen, hydrophobic and ionic bonds) exposes reaction sites and thus improves the efficiency of enzymatic hydrolysis [32,35]. Among the advantages of enzymatic hydrolysis assisted by high hydrostatic pressures are the hydrolysis of proteins normally resistant to enzymatic hydrolysis, the reduction of hydrolysis duration and an increased concentration of peptides, including bioactive peptides [32,36]. Hemker et al. showed that the solubility and emulsifying properties of fish (*Orechromis niloticus*) hydrolysate were increased after enzymatic hydrolysis assisted by high hydrostatic pressures while the water binding capacity was reduced [37]. To the best of our knowledge, no literature is available regarding the effect of high hydrostatic pressure-assisted enzymatic hydrolysis on the functionality of insect hydrolysates. Consequently, the objective of this study is to determine the functional properties of insect meals and insect peptide hydrolysates generated with or without pretreatment of the insect meal with high hydrostatic pressure. This work focuses on crickets (*G. sigillatus*) and mealworms (*T. molitor*), as these insect meals have different nutritional composition and are already produced and sold in Canada.

## 2. Materials and Methods

### 2.1. Materials

#### 2.1.1. Insects

Commercial mealworm (*T. molitor*) and cricket meals (*G. sigillatus*), were purchased from Entomo Farms (Norwood, ON, Canada) and stored at 4 °C. Their proximate compositions are shown in Table 1. Control hydrolysates and protein hydrolysates were produced from each meal by coupling enzymatic hydrolysis and high hydrostatic pressures.

#### 2.1.2. Chemicals

Unless specified, all chemicals used for analytical purposes were analytical grade. Alcalase^®^ (protease from Bacillus licheniformis), β-mercaptoethanol, D-L-Leucine, sodium tetraborate and o-phtaldialdehyde (OPA reagent) were purchased from Sigma Aldrich (St Louis, Missouri, MO, USA). Hexane, hydrochloric acid, methanol and sodium hydroxide were purchased from Fisher Scientific (Ottawa, ON, Canada). Citric Acid monohydrate, hydrochloric acid (36.5–38% *v*/*v*), NaCl, sodium hydroxide pellets and sodium phosphate dibasic were purchased from VWR International (Mississauga, ON, Canada). Sodium dodecyl sulfate (SDS) was purchased from Bio Basic (Markham, ON, Canada). Food grade canola oil was purchased from a local grocery store in Quebec City.

### 2.2. Preparation of Insect Protein Hydrolysates

Control hydrolysate was prepared following the procedure described by Liceaga-Gesualdo and Li-Chan, with some modifications [38]. A mass of 500 g of insect meal was dispersed in deionized water at 5% (*w*/*w*) and magnetically stirred at 4 °C for 12 h. The temperature of dispersion was adjusted to 55 °C and the pH was adjusted at 8.5 with 0.66 N NaOH [39,40]. Alcalase^®^ was then added to the dispersion at 3% (E/S). During the 2 h enzymatic hydrolysis, the dispersion was constantly stirred, and the pH was constantly adjusted with alkaline solution (NaOH, 0.66 N). After hydrolysis, Alcalase^®^ was inactivated by heat treatment at 80 °C for 15 min. The hydrolysate was cooled to 20 °C and centrifuged at 4500× *g* for 45 min at 20 °C. Finally, the supernatant was filtered using a strainer to remove some lipids [41] and freeze-dried. For the high-pressure conditions, both insect dispersions were prepared as described for the control. Pressurization parameters (200 and 380 MPa for 1 min for cricket and mealworm meals, respectively) were based on results obtained by Boukil et al. [42]. All hydrolysates were stored at 4 °C between experiments.

### 2.3. Proximate Composition of Protein Insect Ingredients

Total crude protein content was determined by using the Kjeldahl method according to Association of Official Analytical Chemists (AOAC) 928.08 procedures [43]. Two different conversion factors of 4.76 and 5.60 were used for insect meals and hydrolysates respectively, due to the presence of chitin, a nonprotein nitrogen component in insects [44]. More specifically, the conversion factor of 4.76 was applied for insect meals whereas a conversion factor of 5.60 was used for hydrolysates as suggested by Janssen et al. [44]. Total crude fat content was determined using the Soxhlet extraction method described by Tzompa-Sosa et al. except that hexane was used as the extraction solvent [45]. Chitin content was determined using a gravimetric method based on the method of Spinelli et al. [46]. Chitin is considered the residue after the extraction with 2% (*w*/*v*) sodium hydroxide and demineralization with 5% (*w*/*v*) hydrochloric acid [46]. The standard AOAC methods 950.46A and 920.153 were used to determine dry matter and ash content, respectively [43]. The proximate composition analyses were performed in triplicate except for total crude protein and fat content, which were performed in duplicate.

### 2.4. Degree of Hydrolysis

Freeze-dried insect hydrolysates were rehydrated in deionized water at 5% (*w*/*w*) to determine the degree of hydrolysis. The degree of hydrolysis was determined by the o-phthaldialdehyde (OPA) method according to Church et al. [47]: Different concentrations (0.75–3 mM) of D-L-Leucine were used to obtain the standard curve [48]. The degree of hydrolysis was calculated according to the Equation (1) proposed by Hall et al. [3]:

(1) $$DH\;\left(\%\right)=\left(\frac{h}{h_{tot}}\right)\times 100$$

where *h* is the total peptide bonds cleaved and *h_tot_* is the total peptide bonds. The *h_tot_* of the equation was 8.64 milliequivalents (meq)/g for all samples [3]. Measurements of all samples were performed in triplicate.

### 2.5. Measurement of Particle Size

The distribution of particle sizes for hydrolysates and meals was determined using a laser diffraction system (Mastersizer 3000, Malvern Instrument Ltd., Worcestershire, UK) on hydrolysate and meal dispersions prepared by mixing 0.5 g sample (insect meals and hydrolysates) with 40 mL of McIlvaine buffer (pH 4.0, 5.5 and 7.0) for 12 h at 4 °C. The refraction index of sample dispersion corresponding to the protein refraction index was set to 1.45. The dispersant phase was deionized water and this refraction index was set to 1.33. The dispersion sample was added until an obscuration of approximately 10% was obtained. The particle size distribution was expressed as D3,2 (μm), the sautermean diameter. All conditions were conducted in duplicate.

### 2.6. Protein Solubility

Protein solubility was determined at different concentrations (3.0, 1.0 and 0.5% (*w*/*v*)) of hydrolysates and meals, and at different pHs (4.0, 5.5 and 7.0) by using the methodology described by Morr et al. [49]. Briefly, samples of meals and hydrolysates were dispersed in 50 mL McIlvaine buffer and stirred at 20 °C for 2 h. The dispersion was then centrifuged at 2000× *g* for 30 min at 20 °C. The Kjeldahl method was used to determine the nitrogen content of the supernatants. Protein solubility was calculated using the Equation (2) proposed by Hall et al. [3] and expressed as a percentage:

(2) $$\mathrm{Solubility}\;\left(\%\right)=\;\left(\frac{\mathrm{protein}\;\mathrm{content}\;\mathrm{in}\;\mathrm{supernatant}}{\mathrm{protein}\;\mathrm{content}\;\mathrm{in}\;\mathrm{sample}}\right)\times 100$$

### 2.7. Rheological Behavior

The viscosity of dispersion of the samples was determined using an ARES-G2 rheometer (TA instrument, New Castle, DE, USA) with DIN geometry (diameter 27.77 mm, gap 5.849 mm) with a cup (diameter 29.9 mm). Briefly, 0.75 g of sample (insect meals and hydrolysates) was dispersed at 3% (*w*/*v*) in McIlvaine buffer (pH 4.0, 5.5 and 7.0) in a centrifuge tube and stirred for 30 s. The dispersion was stored at 4 °C for 12 h and stirred again for 30 s. The dispersion was then conditioned at 23 °C and a pre-shear at 10 s^−1^ for 60 s was applied. The flow experiment was performed by increasing the shear rate from 10 to 750 s^−1^. Data were analyzed using TRIOS software (TA Instrument). The flow properties were determined by fitting the data to the Power Law Model on the flow sweep for a shear rate from 10 to 100 s^−1^. Equation (3) describes the Power Law Model:

(3) $$\sigma =k{\dot{y}}^{n}$$

where *σ* is the stress (Pa), *k* is the viscosity (Pa∙s), *y* is the shear rate (s^−1^) and *n* is the flow behavior, allowing fluids classification: pseudoplastic (*n* < 1), dilatant (*n* > 1) and Newtonian (*n* = 1).

### 2.8. Gelation Properties

Visual observation of gel formation was performed according to the method described by Yi et al. [41]. Specifically, 3% and 10% (*w*/*v*) dispersion samples (hydrolysates and insect meals) in McIlvaine buffer (pH 4.0, 5.5 and 7.0) were stirred and stored at 4 °C for 12 h. The dispersion was heated at 86 ± 1 °C in a water bath for 10 min, cooled for 1 min in an ice-water bath and stored at 4 °C for 12 h. Gelation was confirmed if the gel was not deformed when the tube was overturned [41] and the gel formation was determined using dynamic oscillatory measurements. Since no gelation properties were visually observed for hydrolysates, only edible insect meals were tested at 30%, 20% and 10% (*w*/*v*) dispersion in McIlvaine buffer (pH 4.0, 5.5. and 7.0) at two ionic strengths (0 and 1 M NaCl). Measurements were made with an ARES-G2 rheometer (TA Instrument, New Castle, DE, USA) used with DIN geometry (diameter 27.70 mm, gap 5.849 mm) with a cup (diameter 29.9 mm). Two temperature ramps were used, 25 to 85 °C and 85 to 5 °C, at a rate of 5 °C/min. The final temperature of each ramp was kept constant for 10 min. The oscillatory parameters for both temperature ramps were 1.0 Hz for angular frequency and 0.05% for strain. An amplitude step was used with a strain of 0.05% to 500% and angular frequency of 1.0 Hz at 5 °C. Data were analyzed using TRIOS software (TA Instrument). It was considered gel if the elastic behavior (G’) was greater than viscoelastic behavior (G”) [50].

### 2.9. Foaming Properties

The foam capacity was determined by the protocol described by Guo et al.’s [51] method with some modifications: 0.75 g of sample (hydrolysates and insect meals) was dispersed at 3% (*w*/*v*) in McIlvaine buffer (pH 4.0, 5.5 and 7.0) and magnetically stirred at 4 °C for 12 h. The volume of the dispersion was measured in a 100 mL graduated cylinder. The dispersion was transferred to a mixing bowl and aired with an electric hand mixer (KitchenAid) at maximum power for 2 min. Finally, the foam was carefully transferred to the same 100 mL graduated cylinder. The foam capacity was calculated from the Equation (4) of Guo et al. [51]:

(4) $$FC\;\left(\%\right)=\left(\frac{V_{0}-V_{i}}{V_{i}}\right)\times 100$$

where *FC* is the foam capacity, *V*_0_ is the volume of the foam that has been formed and *V_i_* is the volume of the dispersion prior to aeration.

### 2.10. Water and Oil Binding Capacities

Water binding capacity (WBC) was assayed according to Quinn and Paton with some modifications [52]. Briefly, 1 g of each sample (hydrolysates and insect meals) was mixed with 10 g McIlvaine buffer (pH 4.0, 5.5 and 7.0) and vortexed for 30 s. After 10 min at room temperature, samples were centrifuged at 2000× *g* for 30 min at 20 °C. Supernatants were decanted and the residual non-bound water drained by placing the centrifugation tube upside-down on a filter paper for 10 min. WBC was calculated using the Equation (5) of Bußler et al. [11]:

(5) $$WBC\;\left[\frac{g_{water}}{g_{sample,\;DM}}\right]=\frac{m_{0}-m_{1}}{m_{0,DM}}$$

where *m*_0_ is the initial mass of the sample, *m*_1_ is the final mass of the sample and *m*_0,*DM*_ is the initial mass of the sample on dry basis. The oil binding capacity (OBC) was assayed according to the method of Haque and Mozaffar with some modifications [53]. Canola oil (5 g) was added to 1 g of sample (hydrolysates or insect meals). The experimental procedure and calculation of OBC were similar to WBC, except for the addition of a stirring step of 3 × 30 s with 4 min breaks between repetitions. The pellet was weighed immediately after decanting the supernatants. Determination of WBC and OBC were performed in triplicate.

### 2.11. Emulsifying Properties

The spectroturbidimetric procedure of Pearce and Kinsella with the modifications by Liceaga-Gesualdo and Li-Chan was used to determine the emulsifying activity index (EAI) and the emulsion stability index (ESI) [38,54]. Briefly, samples (hydrolysates and insect meals) were dispersed at 3.0%, 1.0% and 0.5% (*w*/*v*) in 100 mL McIlvaine buffer (pH 4.0, 5.5 and 7.0) and stored at 4 °C for 12 h. Then, sample dispersions were mixed with 25 mL of canola oil using an Ultra-Turrax at 13,500 rpm for 1 min and diluted into tubes containing 0.3% SDS solution to reach an absorbance greater than 0.1. Homogeneity of dilution was ensured by inverting the tube six times. Absorbance was read at 500 nm with a UV-visible spectrometer. EAI was calculated from the Equation (6) used by Hall et al. [3]:

(6) $$\mathrm{EAI}=\frac{2\cdot T\cdot A\cdot df}{\emptyset \cdot c\cdot 100}$$

where *T* is the turbidity, *A* is the absorbance measured, *df* is the dilution factor, *L* is the light path (in meters), ∅ is the volume of the oil phase (0.25) and *c* is the concentration of the aqueous phase. The turbidity was calculated according to Pearce and Kinsella [54]. For ESI, the emulsion was read every 30 min for 90 min and was calculated from the equation used 7 by Hall et al. [3]:

(7) $$\mathrm{ESI}=100-\left(\frac{{\mathrm{EAI}}_{0}-{\mathrm{EAI}}_{t}}{{\mathrm{EAI}}_{0}}\right)\times 100$$

where EAI_0_ is the initial EAI (time zero) and EAI_t_ is the EAI at 30, 60 and 90 min.

### 2.12. Statistical Analysis

All analyses were performed in triplicate except for gelling properties (duplicate). A fully random plan was used except for water and oil binding capacities where repetitions were blocked. The factors were insect, treatment, pH, concentration and time. Tukey tests were used as multiple comparison tests with a significance level (α) of 5%. The results were reported as mean ± standard deviation (M ± SD).

## 3. Results and Discussion

### 3.1. Proximate Composition and Degree of Hydrolysis

Table 1 shows the proximate composition of cricket and mealworm meals, as well as their protein hydrolysates generated at atmospheric pressure (control—0.1 MPa) or using high hydrostatic pressures (HHP) treatment prior to enzymatic hydrolysis by Alcalase^®^. Pretreatment of insect meals with HHP did not change the proximate composition of either insect hydrolysate (*p* > 0.05). The dry matter values for all insect species and ingredients (meals and hydrolysates) were close to 100% (96.5 to 98.6%) and slightly higher for mealworms compared to crickets. This difference directly correlates with the differences observed for crude protein, lipid, chitin and ash contents. The chitin content was similar in both insect meals (4.2 to 4.8%) but was significantly lower in hydrolysates (0.02 to 0.07%) because this water-insoluble polysaccharide [40] was removed by the centrifugation step performed to recover the soluble protein fraction. Similarly, lipid content was decreased by about 50% in cricket hydrolysates and 21% in mealworm hydrolysates, compared to their respective initial meals, due to the centrifugation step [41]. The higher lipid concentrations recovered in mealworm (20.6 and 23.3%) vs. cricket hydrolysates (7.5 and 8.3%) were related to differences in fatty acid composition since the unsaturated fatty acid fraction of the mealworm matrix is higher than that in the cricket [45]. The filtration method after the centrifugation step did not successfully remove the entire unsaturated lipid fraction, which was in a liquid form at room temperature. As expected, ash content was higher in control and pressure-treated hydrolysates compared to initial meals for both insect species. This is explained by the addition of Na+ from the NaOH required to control pH during enzymatic hydrolysis [38]. Contrary to previous studies [4,55], the crude protein content was higher for cricket meal (55.5%) than for mealworm meals (39.6%). Insect diet and rearing techniques could account for this difference [56]. After enzymatic hydrolysis, the protein content was increased in both hydrolysates. This result is consistent with the findings of Hall et al. after enzymatic hydrolysis of cricket protein by Alcalase^®^, due to the increase in protein solubility [20,22,23,57]. Table 1 also presents the degree of hydrolysis (DH) after in vitro digestion of cricket and mealworm proteins by Alcalase^®^. The DH was similar for both insect species with values ranging from 28.1 to 33.8%. These values were consistent with values obtained by Boukil et al. [42]: lower than those obtained by Hall et al. [3] after enzymatic hydrolysis of cricket (G. sigillatus) proteins by Alcalase^®^ (ranged from 42.1 to 52.4%; E/S = 3.0%; hydrolysis time ranged from 30 to 90 min) and higher than the published study of Purschke et al. [20], calculated after in vitro digestion of migratory locust proteins with Alcalase^®^ (ranging from 11.6 to 15.2%; E/S = 1.0%; hydrolysis time ranging from 30 to 120 min). Different parameters known to influence the degree of hydrolysis (E/S ratio, temperature, pH and reaction duration) can explain these differences [57,58]. The protein quality is also important since production of edible insect meals at laboratory or commercial scale might impact protein denaturation and aggregation, and consequently, their solubility which could affect the efficiency of enzymatic hydrolysis [19]. As shown in Table 1, HHP pretreatment of mealworm meal decreased the degree of hydrolysis (25.6%) compared to control (33.8%). This result is probably due to protein denaturation and aggregation phenomena caused by HHP which may decrease the efficiency of enzymatic hydrolysis [59].

### 3.2. Particle Size Distribution

Table 2 shows particle size indexes of cricket and mealworm meals at pH 4.0, 5.5 and 7.0, as well as hydrolysates generated with or without pretreatment of insect meals with HHP. As expected, and whatever the pH, higher particle sizes were obtained for both edible insect meals compared to their respective hydrolysates. At pH 4.0, cricket meal particles (D3,2 = 52.7 μm) were bigger than those at pH 5.5 (D3,2 = 47.0 μm) and pH 7.0 (D3,2 = 37.3 μm). The same tendency was observed for cricket hydrolysates. Since pH 4.0 is close to the isoelectric point (pI) of commercial cricket meal (pI close to 3.85) [60], the repulsive forces between the proteins decrease, inducing their aggregation [61]. Surprisingly, the particle size (D3,2) of mealworm meal increased with increasing pH (4.0–7.0) (values ranging from 17.0 to 41.4 μm), whereas the pI of the proteins was 3.95 [60]. This tendency was the same for mealworm hydrolysates and could be explained by the formation of disulfide bonds between mealworm proteins or even by the formation of protein–lipid complexes [62]. For both insects, the pretreatment of meals with HHP did not modify the particle sizes.

### 3.3. Solubility of Insect Meals and Hydrolysates

Table 3 shows the protein solubility of cricket and mealworm meals and their hydrolysates (control and generated from pressure-treated insect meals) at different pH values (4.0, 5.5 and 7.0) and concentrations (0.5–3.0% *w*/*v*). The sample concentration (0.5–3.0% *w*/*v*) and pH values (4.0, 5.5 and 7.0) modified the protein solubility of insect meals. Regardless of pH and concentration, cricket and mealworm meals had low solubility with values ranging from 17.1 to 18.7% and from 15.8 to 20.2%, respectively. These values are consistent with those available in the literature [3,11,61,63]. More specifically, Stone [60] obtained protein solubility ranging from 29.0 to 23.4% between pH 3.0 and 7.0 for commercial cricket and mealworm meals. A study published by Kröncke et al. confirmed that oven drying of *T. molitor* larvae decreased the quality of proteins and reduced their solubility by 74% [19]. This low solubility was mainly related to the drying method (oven-drying) applied at commercial scale before the larvae grinding step. The heat treatment denatured the protein, exposing hydrophobic groups and causing protein aggregation [21,64].

The protein solubility of hydrolysates, which was similar for all pHs (4.0–7.0) and sample concentrations (0.5–3.0% *w*/*v*), was drastically improved compared to insect meals, with values ranging from 92.5 to 100.0% and 72.9 to 78.9%, for cricket and mealworm hydrolysates, respectively. This improvement in solubility is consistent with results obtained by Hall et al. and Wang et al. for cricket and house fly larvae after enzymatic hydrolysis by Alcalase^®^ [3,24]. The digestion of protein into peptides increases ionizable groups, such as amino and carboxyl groups, improving hydration [65] and solubility. Furthermore, the higher solubility was also related to the fact that only the soluble fraction was freeze-dried during hydrolysate preparation [22].

Compared to the control, pressurization pretreatment of cricket meal slightly decreased the hydrolysate solubility, mainly at 1.0 and 3.0% for pH 4.0, 5.5 and 7.0 (Table 3). Protein solubility is influenced by the type of protein, protein concentration, pH and the presence of salts [66]. However, the similar compositions (Table 1) and particle sizes (Table 2) could not explain the differences in solubility between control and pressure-treated hydrolysates for both insect species. Gbogouri et al. [57], studying protein hydrolysate from salmon, showed that hydrolysates with a higher degree of hydrolysis generally had higher solubilities than those possessing lower DH. Indeed, smaller peptides from hydrolysates with high DH were considered to have more polar residues, which could enhance the quantity of hydrogen bonds with water, resulting in an increase in protein solubility in solution [37,67]. However, the similar DH values calculated for cricket hydrolysate (control and pressure treatments) (Table 1) could not explain their differences in solubility. HHP parameters, such as pressure, duration and temperature, also impact protein solubility. Under pressure, the loss of solubility is mainly related to the formation of insoluble high molecular weight protein aggregates due to exposure of hydrophobic residues and/or disulfide bond formation [68,69]. Consequently, during the enzymatic hydrolysis of pressure-treated protein, enzymes break the protein in different ways due to modifications of protein structure since some bonds became inaccessible to the enzymes and, on the contrary, others may be exposed due to conformational changes [36,70,71]. Therefore, Alcalase^®^ hydrolysis of cricket meal pretreated by pressure could generate a different peptide profile that contains more hydrophobic peptides, which could negatively impact hydrolysate solubility. On the contrary, the solubility of mealworm hydrolysate generated from pressure-treated meals increased for all concentrations and pH values (Table 3). Kim et al., studying hemoglobin hydrolysate, mentioned that the hydrolysate solubility could be improved at low DH due to the generation of hydrophobic peptides in lower amounts [72]. Consequently, and as proposed for cricket hydrolysate, the protein unfolding and aggregation due to pressurization could generate a different peptide profile with a larger amount of hydrophilic peptides, improving hydrolysate solubility.

### 3.4. Rheological Behavior

Table 4 shows the viscosity and the flow behavior rate of cricket and mealworm meals and their hydrolysates (control and generated from pressure-treated insect meals) at different pHs (4.0, 5.5 and 7.0). Whatever the pH and the treatment (meals and hydrolysates), the viscosity of insect meals and hydrolysates for all conditions analyzed was very near 0 Pa∙s. The viscosity of cricket and mealworm meals (0.7 mPa∙s) was slightly lower than hydrolysates (1.2 mPa∙s) due to the insoluble particles of insect meals which settle quickly, resulting in the rheological behavior of only the soluble phase being measured. Generally, the viscosity of hydrolysates is lower than the initial proteins due to the small sizes of the peptides [73,74,75]. For both insects, treating insect meals with HHP prior to enzymatic digestion did not change the viscosity compared to the control hydrolysate. Whatever the pH and treatment (meals and hydrolysates), the flow behavior rate was around 1, which indicated that all samples behaved as Newtonian fluids. Thus, the insect, treatment or pH did not modify the flow behavior rate (*p* > 0.05). Jung et al. have shown that the flow behavior index values change from pseudoplastic (*n* < 1) to Newtonian behavior (*n* = 1) after the enzymatic digestion of soy protein [73].

### 3.5. Gelation Properties

Gel formation was not observed with insect meals and hydrolysates (control and generated from pressure-treated insect meals) at 3% (*w*/*v*) or 10% (*w*/*v*), which is probably caused by the denaturation of proteins during the drying method used for commercial production of insect meals. Hydrolysates are also generally known to have poor gelling properties [58,74]. Protein concentration is a key factor influencing gelation properties and the gelation threshold depends on structural characteristics and gelling conditions such as pH and ionic strength [76,77,78]. Therefore, the concentrations used (3 and 10% *w*/*v*) in our study were probably insufficient to reach the gelation threshold.

**Figure 1:**
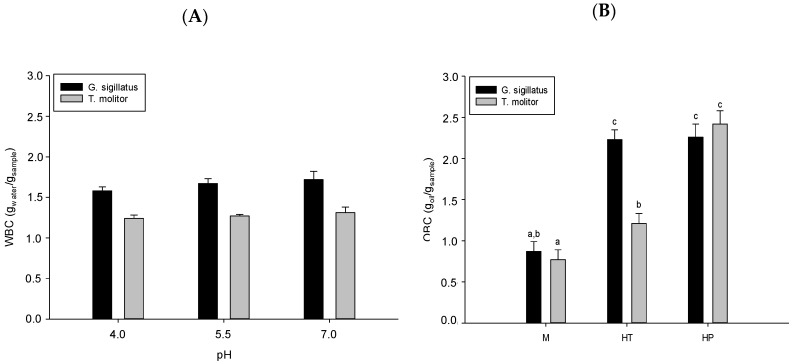
Water binding capacity (**A**) and oil binding capacity (**B**) of insect meals (M) and hydrolysates (HT: control hydrolysate, HP: hydrolysate generated by HHP treatment of meal prior to enzymatic hydrolysis). Different letters indicate significant difference (*p* < 0.05).

**Figure 2:**
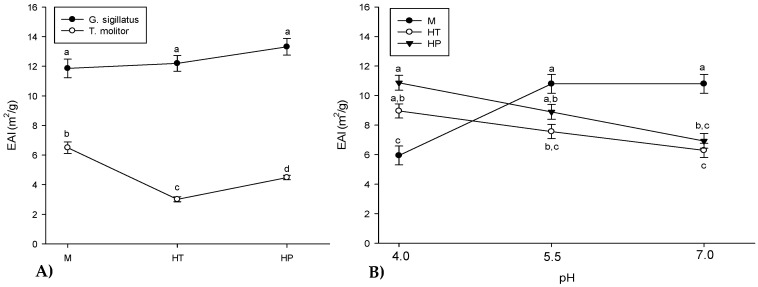
Emulsion activity index (EAI) of insect meals (M) and hydrolysates (HT: control hydrolysate, HP: hydrolysate generated by HHP treatment of meal prior to enzymatic hydrolysis) according to the insect source (cricket and mealworms) (**A**) and according to the pH (4.0–7.0) (**B**). Different letters indicate significant difference (*p* < 0.05).

The gelation abilities of higher concentrations of insect meals were evaluated using dynamic rheology. The results were similar for both insect meals regardless of the experimental conditions (10, 20 and 30% *w*/*v* at pH 7.0, with or without 1M NaCl). The rheological behavior of cricket meal is given as an example (Figure 1A). For cricket and mealworm meals, the elasticity modulus (G’) was higher than the loss modulus (G”), which indicates that gelation occurred, regardless of pH and ionic strength. However, the higher G’ values that should indicate gelation were observed before heat treatment, suggesting that a protein gel may not be responsible for this rheological behavior. The insect meals had very low solubility (15.84–20.17%, Table 3) and the insoluble particles settled quickly after agitation. In addition, certain ionic strengths could not be analyzed because the maximum axial force of the device was reached before the analysis could be performed, also suggesting that a packed precipitate formed at the bottom of the geometry. To confirm that no gelation occurred, the experimental conditions described at the beginning of this section were reproduced in glass tubes. Two distinct phases (soluble and insoluble phases) were observed (Figure 2A). The texture of the insoluble phase was similar to wet sand while the upper phase was liquid. After heat treatment, the two phases had a similar texture, but the proportion of insoluble phase seemed to increase compared to the soluble fraction (Figure 2A). From these observations, the changes in rheological behavior observed (in Figure 1A) could be explained by temperature-induced modification of the physico-chemical characteristics of the precipitated phase. Consequently, G’ and G” moduli decreased during the heating phase and increased during the cooling phase, which is representative of protein interactions where the hydrophobic interactions increase with increasing temperature and hydrogen bonds are favored with cooling [79,80]. Zhao et al. [61] also observed this phenomenon for a mealworm protein concentrate. Moreover, the G’ and G” moduli were slightly lower until the cooling step and similar after this step. These authors demonstrated that the effect of the viscoelastic moduli of the salt concentration was pH-dependent [81]. In the literature, only few studies mention gelation properties of insect proteins [41,61]. More specifically, Yi et al. [41] obtained gelation of an *A. domesticus* soluble fraction (3% *w*/*v*) at pH 7.0 and gelation of different insect meal soluble fractions (*T. molitor, A. diaperinus, Z. morio* and *B. dubia*), but at high concentration (30% *w*/*v*) for pH 7.0 and 5.0. Otherwise, the authors generally obtained only an aggregation induced by the heat treatment [41]. Zhao et al. [81] obtained a weak gel after adding 2% NaCl to a mealworm protein concentrate since NaCl can improve the gelation properties of proteins by reducing the repulsive forces between proteins.

### 3.6. Foaming Properties

No foaming properties could be measured for cricket and mealworm meals and hydrolysates (control and generated after HHP), mainly due to foam destabilization during aeration. Several studies have shown that insect proteins have poor foaming properties [41,63,82,83]. Stone obtained a foaming capacity of around 82% for a commercial cricket meal but no foaming properties were observed for commercial mealworm meal [60]. According to Hall et al., enzymatic digestion enhanced the foam capacity of cricket proteins due to structural and conformational modifications [3]. Enzymatic hydrolysis generates low molecular weight peptides and exposes surface-stabilizing residues at the air–water surface which can allow rapid migration, better flexibility and rearrangement at the interface and, therefore, improve foaming properties [38,58]. In our study, the poor foaming properties of the different insect ingredients is mainly due to their lipid content. Indeed, it is well-known that just 0.5% lipid can reduce the volume of foam and cause destabilization during aeration of egg whites [84]. Consequently, efficient defatting of insect meals is crucial to generate a food ingredient with good foaming properties.

### 3.7. Water and Oil Binding Capacities

Figure 1A shows the WBC of cricket and mealworm meals at pH 4.0, 5.5 and 7.0. The simple effect of insect (crickets and mealworms) and pH (4.0, 5.5 and 7.0) variation was significant (*p* < 0.0001), but their interactions were not (*p* = 0.08). WBC was calculated for cricket and mealworm hydrolysates (control and generated from pressure-treated insect meals) since their composition of soluble peptides did not retain water. However, Purschke et al. [20] obtained WBC values for commercially migratory locust hydrolysates (close to 1.50 g_water_/g) after enzymatic hydrolysis with Neutrase or Flavourzyme. This can be explained by differences in the composition of edible insects, experimental conditions and hydrolysis parameters. As observed in Figure 2A, the WBC of cricket meal (1.58–1.72 g_water_/g) was higher than mealworm meal (1.24–1.31 g_water_/g), probably because mealworms have a lower hydrophilic amino acid content than crickets [55]. Our values for commercial cricket and mealworm meals (1.76 g_water_/g and 1.62 g_water_/g, respectively) were consistent with those published by Stone [60]. The WBC of insects is generally lower than several vegetable proteins such as soy protein isolate (4.47 g_water_/g), red kidney beans (2.25–2.65 g_water_/g) and Indian kidney beans (2.60 g_water_/g) [85]. Many factors influence the WBC, such as the amino acid profile, conformation, hydrophobicity, pH, ionic strength, temperature and protein concentration [86]. The water binding capacity at pH 4.0 was significantly (*p* < 0.05) lower than at pH 5.5 or 7.0. As previously mentioned, the pI of commercial cricket and mealworm meals is close to 3.85 and 3.95, respectively. The pH affects the charge on proteins and, consequently, close to the pI, the WBC is minimal since protein–protein interactions are favored over protein–water interactions [86].

Figure 1B also shows the OBC of insect meals and hydrolysates (control and generated from pressure-treated insect meals). The OBC was similar for both insect meals with values ranging from 0.77 to 0.87 g_oil_/g, but lower than the results obtained by Stone [60] for commercial cricket and mealworm meals (1.42 g_oil_/g and 1.58 g_oil_/g, respectively) and by Purschke et al. [20] for commercial migratory locust meals using rapeseed oil (1.10 g_oil_/g). These differences may be due to the use of different oils, but many other factors such as the amino acid composition and degree of denaturation of the proteins may also have had an effect [85]. Similar to WBC, the insect OBC is generally lower than for other proteins, such as soy protein isolate (1.54 g_oil_/g), red kidney beans (1.23–1.52 g_oil_/g) and Indian kidney beans (2.40 go_il_/g) [85]. The OBC of control hydrolysates increased from 0.87 to 2.23 g_oil_/g and from 0.77 to 1.21 g_oil_/g for cricket and mealworms, respectively. This tendency, also observed by Purschke et al. [20] for migratory locust protein following enzymatic hydrolysis by Neutrase and Flavourzyme (alone or in combination), is due to the exposure of hydrophobic groups that were previously hidden in the edible insect protein structure [20,87]. While HHP did not impact the OBC of cricket hydrolysate, it doubled OBC for mealworm hydrolysate compared to the control (1.21 to 2.42 g_oil_/g). The decrease in the degree of hydrolysis between control and HHP mealworm hydrolysates possibly explains this tendency, as Chalamaiah et al. [22] showed that a higher degree of hydrolysis reduces oil binding capacity. The HHP treatment probably modified the protein structures and peptide profile due to different enzyme breaks.

### 3.8. Emulsifying Properties

#### 3.8.1. Emulsion Activity Index (EAI)

Figure 2A shows the EAI values of cricket and mealworm meals and their hydrolysates (control and generated from pressure-treated insect meals). Higher EAI values mean that the dispersed fat droplets are smaller in size and that proteins (or peptides) have more ability to absorb at the oil–water interface [88]. Globally, cricket ingredients (11.86–13.32 m^2^/g) had higher EAI values compared to mealworms (3.01–6.50 m^2^/g) which could be due to the difference in the hydrophobicity of the proteins and the smaller size of cricket proteins, which would allow faster diffusion at the oil–water interface [23,57,89]. Chatsuwan et al. [90] obtained EAI values ranging from 29.23 to 36.69 m^2^/g for *P. succinta* and *C. rosea*, respectively. Regardless of the insect species, the lower EAI values in our study can be attributed to the drying method used to produce commercial insect meals, which modifies protein solubility [19] and emulsifying properties [91]. The EAI (*p* > 0.05) of the cricket hydrolysate control was not significantly different from the meal. This tendency was also observed by Hall et al. for some enzymatic hydrolysis conditions of cricket using Alcalase^®^ (E/S of 0.5%; hydrolysis time of 90 min and E/S of 1.5%; hydrolysis time of 30 min) compared to unhydrolyzed proteins [3]. Conversely, the EAI value of the mealworm control hydrolysate was reduced compared to the meal. Hall et al. also obtained a decreased EAI after enzymatic hydrolysis of tropical banded cricket with similar enzymatic digestion conditions (E/S of 3%; hydrolysis time of 90 min) [3]. This reduction of EAI was possibly caused by the enzymatic hydrolysis parameters which would have reduced the interfacial activity of proteins due to excessive protein degradation [57,92]. However, several studies have shown that the enzymatic hydrolysis of several proteins using Alcalase^®^ improved their EAI values [23,38,88]. This has been explained by the enzyme’s specificity in cutting aromatic residues to reveal hydrophobic peptides, which facilitates the formation of emulsions [73,93]. In addition, the increased peptide solubility after enzymatic hydrolysis promotes peptide absorption at the oil–water interface [23]. Applying HHP treatment prior to enzymatic hydrolysis had no impact on EAI values for cricket, since the degree of hydrolysis was similar. However, HPP treatment of the mealworm meal increased its EAI compared to the control hydrolysate, possibly due to modification of the peptide hydrophobicity [89] caused by different enzyme cut sites linked to the structural modifications of proteins after HHP. Despite this improvement of EAI, the value is still lower than unhydrolyzed mealworm meal.

Figure 2B shows the EAI of insect ingredients (meals and hydrolysates) at different pHs (4.0, 5.5 and 7.0), regardless of insect and concentration of the aqueous phase. The insect meals had significantly (*p* < 0.05) lower EAIs at pH 4.0 than at pH 5.5 and 7.0, near the pI of commercial insect meals (3.85 and 3.95 respectively, for cricket and mealworm meals) [60]. Measuring at the pI produced the lowest EAI (Table 3), consequently, diffusion and adsorption of protein at the oil–water interface were delayed [94]. This reduction of EAI at the pI was also observed for chickpea and whey protein isolate [95,96]. For hydrolysates (control and HHP-treated), the EAI decreased slightly with increasing pH (4.0–7.0) due, once again, to solubility modification. Pacheco-Aguilar et al. [88] obtained similar EAI values for fish (*Mercuccius productus*) protein hydrolysate at pHs 4.0 and 7.0, but higher values at pH 10.0 and a different degree of hydrolysis (DH 10–20%).

#### 3.8.2. Emulsion Stability Index (ESI)

Table 5 shows the ESI of cricket and mealworm meals and hydrolysates (control and from pressure-treated insect meals), and different concentrations of aqueous phase (0.5–3.0% *w*/*v*) and pH (4.0–7.0). Significant triple interactions were obtained between the different treatments (meals and hydrolysates), concentrations (0.5, 1.0 and 3.0% *w*/*v*) and times (30, 60 and 90 min) (*p* < 0.0001) as well as insects, treatments and time (*p* < 0.01) (Table A1). Specifically, the variable time is an influencing factor. Indeed, the emulsions were very unstable since most of the destabilization process (28.8–59.5% and 39.1–76.6% for meals and hydrolysates, respectively) occurred within 30 min. A creaming phenomenon has been observed only a few minutes after the homogenization was stopped. The ESI values obtained by Hall et al. [3] after 30 min for cricket hydrolysate (E/S of 0.5–3%; hydrolysis time of 30–90 min) were generally below 60%. Several studies have shown that the emulsion stability of insect proteins was generally low (21.2–45.4%) due to their very low solubility [63,85,90]. The size of proteins (or peptides) and the amino acid composition and distribution also influence the strength of the interfacial layer and therefore the emulsion stability [3,88]. Enzymatic hydrolysis slightly enhanced the ESI values for cricket but did not seem to affect the mealworms (Table 5). For example, using a cricket concentration of 3.0% (*w*/*v*), pH 7.0 and 30 min treatment resulted in ESI values ranging from 51.5 to 72.1% after enzymatic digestion (with or without pressure-treated meals), while the ESI ranged from 59.5 to 53.0–55.4 for mealworms. Hydrolysis using Alcalase^®^ has been shown to improve emulsion stability in different studies [3,38]. This might be caused by stronger and more cohesive interfacial layer films with peptides compared to initial proteins [38]. For both insects, the treatment of insect meals by HHP prior to the enzymatic digestion did not change the ESI values. For example, the ESI values at a concentration of 3.0% (*w*/*v*) and pH 7.0 ranged from 67.23 to 72.1% and 53.0 to 55.4 for crickets and mealworms, respectively. For both insects, a concentration of 3% (*w*/*v*) produced better stability than other concentrations analyzed (0.5–1.0%). The effect of pH and concentration was similar for hydrolysates generated at atmospheric pressures or by pressure-treated insect meals. Bußler et al. [11] observed that insect emulsion stability decreased near the isoelectric point due to weak electrostatic repulsion between the oil droplets, but this phenomenon was not observed in this study.

## 4. Conclusions

This study demonstrated that some functional properties of commercial insect meals, mainly solubility and the OBC, can be enhanced by enzymatic hydrolysis by Alcalase^®^. More specifically, the WBC and the foaming and gelation properties were not improved by enzymatic digestion (control and pressure-treated insect meals). Moreover, meals and hydrolysates had low emulsifying properties and viscosity. These results corroborate their current use as filler agents to produce protein-enriched bread and pasta. The DH was further improved by the use of HHP, but only for the mealworms. Despite only a moderate effect of HHP, these results showed the potential of insect protein hydrolysate as a new food ingredient for food formulations. However, to produce insect-based ingredients with better functionalities, it is crucial to control the heating step during insect drying to minimize its negative impact on protein functionality while ensuring microbiological safety of the insect ingredients. The use of extrusion-cooking technology on insect meals and hydrolysates also represents an advantage for improving their functional properties.

## Figures and Tables

**Table 1:** Proximate composition and degree of hydrolysis of cricket and mealworm meals and hydrolysates generated at atmospheric pressure or with the pretreatment of insect meals with high hydrostatic pressures (HHP) prior to enzymatic hydrolysis.

Insects	Treatments ^1^	Crude Protein	Lipid	Chitin ^2^	Dry Matter ^2^	Ash	Degree of Hydrolysis
		% *w*/*w* (dry basis)					
*G. sigillatus*	M	55.5 ± 0.3 ^b^	16.7 ± 0.1 ^c^	4.8 ± 0.2 ^A^	98.0 ± 0.1 ^a, A^	4.8 ± 0.1 ^d^	0
	HT	70.0 ± 0.4 ^a^	7.5 ± 0.1 ^d^	0.03 ± 0.01 ^B^	96.5 ± 0.4 ^a, B^	13.4 ± 0.1 ^a^	28.1 ± 4.2 ^a, b^
	HP	68.2 ± 1.1 ^a, b^	8.3 ± 0.6 ^d^	0.02 ± 0.02 ^B^	97.5 ± 0.2 ^a, C^	13.1 ± 0.1 ^a^	29.6 ± 1.3 ^a, b^
*T. molitor*	M	39.6 ± 4.4 ^c^	36.8 ± 0.3 ^a^	4.1 ± 0.6 ^A^	98.6 ± 0.1 ^b, A^	3.3 ± 0.0 ^e^	0
	HT	59.6 ± 4.3 ^a, b^	23.3 ± 0.2 ^b^	0.06 ± 0.02 ^B^	97.0 ± 0.1 ^b, B^	9.3 ± 0.1 ^c^	33.8 ± 1.5 ^a^
	HP	67.5 ± 5.1 ^a, b^	20.6 ± 2.1 ^b, c^	0.07 ± 0.07 ^B^	97.7 ± 0.2 ^b, C^	9.8 ± 0.1 ^b^	25.5 ± 2.8 ^b^

^1^ M: insect meal, HT: control hydrolysate, HP: Hydrolysate whose insect meal has been pretreated at high hydrostatic pressures before enzymatic digestion. Values, except crude protein, represent the mean of three replicates ± standard deviation. Results with different letters are significantly different (*p* < 0.05). ^2^ Interaction was not significant, so lower-case letters (^a^, ^b^, ^c^, ^d^, ^e^) represent insect effect on results and upper-case letters (^A^, ^B^, ^C^) represent treatment effect on results.

**Table 2:** Particle size distribution indexes of cricket and mealworm meals and hydrolysates generated with or without HHP pretreatment of meals.

Insects	Treatments	pH	D(3,2) ^1,2^ μm
*G. sigillatus*	M	4.0	52.7 ± 15.1 ^a, A^
		5.5	47.0 ± 1.6 ^a, A, B^
		7.0	37.3 ± 3.3 ^a, B^
	HT	4.0	7.6 ± 5.3 ^b, A^
		5.5	0.21 ± 0.03 ^b, A, B^
		7.0	0.09 ± 0.00 ^b, B^
	HP	4.0	6.4 ± 7.5 ^b, A^
		5.5	0.69 ± 0.72 ^b, A, B^
		7.0	0.11 ± 0.01 ^b, B^
*T. molitor*	M	4.0	17.0 ± 3.1 ^a, A^
		5.5	37.3 ± 8.8 ^a, A, B^
		7.0	41.2 ± 17.6 ^a, B^
	HT	4.0	5.8 ± 1.9 ^b, A^
		5.5	4.9 ± 0.3 ^b, A, B^
		7.0	6.2 ± 1.1 ^b, B^
	HP	4.0	5.0 ± 1.7 ^b, A^
		5.5	5.5 ± 0.8 ^b, A, B^
		7.0	6.4 ± 1.4 ^b, B^

M: insect meal, HT: control hydrolysate, HP: Hydrolysate generated by the treatment of insect meal with high hydrostatic pressures prior to enzymatic hydrolysis, D(3,2): area-based mean particle diameter. Values represent the mean of three replicates ± standard deviation. ^1^ Results with different letters within an insect are significantly different (*p* < 0.05). ^2^ Lower-case letters (^a^, ^b^) represent insect–treatment interaction and upper-case letters (^A^, ^B^) represent insect–pH interaction.

**Table 3:** Solubility of cricket and mealworm meals and hydrolysates at different concentrations and pH values, generated with or without HHP pretreatment before enzymatic digestion.

Insects	Treatment	Concentration (% *w*/*v*)	pH	Solubility (%)
*G. sigillatus*	M	0.5	4.0	17.9 ± 0.6 ^a^
			5.5	18.5 ± 0.6 ^a^
			7.0	18.7 ± 0.6 ^a^
		1.0	4.0	17.1 ± 0.6 ^a^
			5.5	18.7 ± 0.6 ^a^
			7.0	18.6 ± 0.6 ^a^
		3.0	4.0	17.1 ± 0.6 ^a^
			5.5	18.5 ± 0.6 ^a^
			7.0	17.9 ± 0.6 ^a^
	HT	0.5	4.0	98.1 ± 0.9 ^a, b, c^
			5.5	96.7 ± 0.9 ^a, b, c^
			7.0	98.7 ± 0.9 ^a, b^
		1.0	4.0	100.2 ± 0.1 ^a^
			5.5	94.4 ± 0.9 ^c, d^
			7.0	92.5 ± 0.9 ^d^
		3.0	4.0	95.8 ± 0.9 ^b, c, d^
			5.5	95.5 ± 0.9 ^b, c, d^
			7.0	95.6 ± 0.9 ^b, c, d^
	HP	0.5	4.0	92.9 ± 0.9 ^a^
			5.5	92.4 ± 0.9 ^a^
			7.0	90.9 ± 0.9 ^a^
		1.0	4.0	94.7 ± 0.9 ^a^
			5.5	91.4 ± 0.9 ^a^
			7.0	92.9 ± 0.9 ^a^
		3.0	4.0	92.5 ± 0.9 ^a^
			5.5	92.1 ± 0.9 ^a^
			7.0	92.1 ± 0.9 ^a^
*T. molitor*	M	0.5	4.0	16.2 ± 1.0 ^b^
			5.5	17.2 ± 1.0 ^a, b^
			7.0	17.1 ± 1.0 ^a, b^
		1.0	4.0	15.8 ± 1.0 ^b^
			5.5	17.5 ± 1.0 ^a, b^
			7.0	18.1 ± 1.0 ^a, b^
		3.0	4.0	16.8 ± 1.0 ^a, b^
			5.5	16.1 ± 1.0 ^b^
			7.0	20.2 ± 1.0 ^a^
	HT	0.5	4.0	75.7 ± 1.9 ^a^
			5.5	73.1 ± 1.9 ^a^
			7.0	72.9 ± 1.9 ^a^
		1.0	4.0	77.0 ± 1.9 ^a^
			5.5	79.0 ± 1.9 ^a^
			7.0	74.8 ± 1.9 ^a^
		3.0	4.0	73.4 ± 1.9 ^a^
			5.5	73.4 ± 1.9 ^a^
			7.0	76.7 ± 1.9 ^a^
	HP	0.5	4.0	108.7 ± 1.5 ^a^
			5.5	88.6 ± 1.5 ^b, c^
			7.0	87.9 ± 1.5 ^b, c^
		1.0	4.0	89.3 ± 1.5 ^b, c^
			5.5	94.2 ± 1.5 ^a, b^
			7.0	88.9 ± 1.5 ^b, c^
		3.0	4.0	88.2 ± 1.5 ^b, c^
			5.5	89.1 ± 1.5 ^b, c^
			7.0	86.7 ± 1.5 ^c^

M: insect meal, HT: control hydrolysate, HP: Hydrolysate generated by HHP treatment of insect meal prior to enzymatic hydrolysis. Values represent the mean of three replicates ± standard deviation. Results in the same insects-treatment with different letters (^a^, ^b^, ^c^, ^d^) are significantly different (*p* < 0.05).

**Table 4:** Viscosity and flow behavior rate of insect meals and hydrolysates at different pHs, generated with or without HHP treatment prior to enzymatic digestion.

Insects	Treatments	pH	Viscosity	Flow Behavior Rate
			mPa∙s	
*G. sigillatus*	M	4.0	0.52 ± 0.53 ^a^	1.26 ± 0.23 ^a^
		5.5	0.84 ± 0.50 ^a^	1.13 ± 0.15 ^a^
		7.0	0.90 ± 0.83 ^a^	0.83 ± 0.58 ^a^
	HT	4.0	1.14 ± 0.18 ^b^	1.07 ± 0.04 ^a^
		5.5	1.23 ± 0.04 ^b^	1.08 ± 0.03 ^a^
		7.0	1.33 ± 0.10 ^b^	1.05 ± 0.04 ^a^
	HP	4.0	1.08 ± 0.21 ^b^	1.06 ± 0.03 ^a^
		5.5	1.04 ± 0.16 ^b^	1.05 ± 0.01 ^a^
		7.0	1.29 ± 0.22 ^b^	1.05 ± 0.02 ^a^
*T. molitor*	M	4.0	0.66 ± 0.55 ^a^	1.31 ± 0.44 ^a^
		5.5	0.68 ± 0.53 ^a^	1.23 ± 0.28 ^a^
		7.0	0.43 ± 0.66 ^a^	1.44 ± 0.36 ^a^
	HT	4.0	1.20 ± 0.05 ^b^	1.04 ± 0.02 ^a^
		5.5	1.10 ± 0.14 ^b^	1.07 ± 0.04 ^a^
		7.0	1.22 ± 0.00 ^b^	1.03 ± 0.01 ^a^
	HP	4.0	1.21 ± 0.09 ^b^	1.04 ± 0.01 ^a^
		5.5	1.11 ± 0.22 ^b^	1.07 ± 0.03 ^a^
		7.0	1.40 ± 0.03 ^b^	1.04 ± 0.00 ^a^

M: insect meal, HT: control hydrolysate, HP: Hydrolysate generated by HHP treatment of insect meal prior to enzymatic hydrolysis. Values represent the mean of three replicates ± standard deviation. Results with different letters (^a^ and ^b^) are significantly different (*p* < 0.05).

**Table 5:** ESI of cricket and mealworm meals and their hydrolysates (control and generated from pressure-treated insect meals) according to the aqueous phase concentration and pH.

Insects	Treatments	Concentration (% *w*/*v*)	pH	ESI (%)		
				30 min	60 min	90 min
*G. sigillatus*	M	0.5	4.0	34.5 ± 1.6	33.1 ± 1.7	31.6 ± 1.4
			5.5	35.0 ± 3.6	32.2 ± 2.3	31.4 ± 3.3
			7.0	33.7 ± 1.8	32.0 ± 2.1	30.4 ± 1.8
		1.0	4.0	35.4 ± 4.4	33.7 ± 3.2	30.1 ± 2.4
			5.5	28.8 ± 7.3	26.4 ± 6.6	24.6 ± 6.7
			7.0	36.6 ± 3.6	33.2 ± 2.9	31.4 ± 3.2
		3.0	4.0	51.5 ± 2.6	43.7 ± 3.5	42.2 ± 1.7
			5.5	50.1 ± 4.0	43.8 ± 1.5	40.7 ± 3.1
			7.0	51.4 ± 0.8	44.7 ± 3.1	41.1 ± 1.1
	HT	0.5	4.0	49.6 ± 3.4	47.3 ± 2.3	45.3 ± 0.8
			5.5	46.6 ± 2.8	45.1 ± 1.3	43.3 ± 1.6
			7.0	48.8 ± 2.2	47.0 ± 1.8	45.0 ± 1.1
		1.0	4.0	59.1 ± 0.6	54.7 ± 3.1	52.8 ± 1.7
			5.5	57.4 ± 2.3	52.5 ± 2.0	49.9 ± 1.1
			7.0	56.2 ± 2.8	53.8 ± 1.9	51.6 ± 3.3
		3.0	4.0	74.8 ± 2.1	71.8 ± 2.7	68.0 ± 3.0
			5.5	75.1 ± 0.9	69.1 ± 2.8	65.7 ± 2.8
			7.0	67.3 ± 1.7	63.8 ± 4.0	61.0 ± 2.1
	HP	0.5	4.0	42.1 ± 4.5	39.4 ± 5.9	38.0 ± 6.9
			5.5	39.5 ± 5.8	36.3 ± 6.6	35.6 ± 7.3
			7.0	40.3 ± 6.4	37.1 ± 7.3	36.0 ± 7.9
		1.0	4.0	60.5 ± 2.0	55.9 ± 2.2	49.9 ± 1.2
			5.5	59.0 ± 7.9	54.1 ± 4.2	49.3 ± 2.0
			7.0	50.9 ± 2.2	47.5 ± 1.7	45.1 ± 2.9
		3.0	4.0	76.6 ± 1.2	70.4 ± 4.9	67.8 ± 5.3
			5.5	74.6 ± 1.5	69.1 ± 2.2	65.5 ± 2.7
			7.0	72.1 ± 1.7	64.3 ± 2.5	64.0 ± 1.2
*T. molitor*	M	0.5	4.0	53.1 ± 2.4	50.9 ± 3.2	50.0 ± 3.2
			5.5	7.0 ± 3.8	43.8 ± 3.2	41.3 ± 3.3
			7.0	46.01 ± 1.0	41.9 ± 0.9	39.7 ± 0.5
		1.0	4.0	46.8 ± 10.9	44.0 ± 9.3	41.7 ± 9.5
			5.5	44.2 ± 5.7	40.4 ± 4.1	36.0 ± 4.3
			7.0	48.1 ± 1.5	42.8 ± 1.4	38.5 ± 2.0
		3.0	4.0	54.6 ± 6.4	48.8 ± 6.5	45.4 ± 6.1
			5.5	55.9 ± 2.1	49.2 ± 4.0	43.7 ± 4.5
			7.0	59.5 ± 3.7	51.2 ± 3.1	46.2 ± 4.3
	HT	0.5	4.0	48.9 ± 0.3	47.3 ± 1.0	46.7 ± 0.9
			5.5	40.5 ± 4.7	38.5 ± 6.7	38.2 ± 6.7
			7.0	39.1 ± 5.4	38.6 ± 5.4	37.5 ± 5.6
		1.0	4.0	44.9 ± 8.7	42.7 ± 10.0	42.9 ± 8.8
			5.5	46.9 ± 4.8	44.9 ± 5.4	44.0 ± 5.3
			7.0	47.9 ± 1.6	45.1 ± 2.5	44.3 ± 2.8
		3.0	4.0	58.9 ± 6.4	53.1 ± 5.7	51.1 ± 6.6
			5.5	51.2 ± 4.5	48.3 ± 5.9	46.9 ± 4.89
			7.0	53.0 ± 6.9	49.4 ± 7.4	46.6 ± 6.3
	HP	0.5	4.0	39.1 ± 4.8	38.5 ± 5.1	37.3 ± 4.7
			5.5	41.1 ± 0.9	39.8 ± 1.6	38.8 ± 0.8
			7.0	40.5 ± 0.7	39.2 ± 1.2	37.8 ± 0.8
		1.0	4.0	46.5 ± 1.4	42.4 ± 2.7	40.6 ± 3.4
			5.5	46.4 ± 5.4	43.2 ± 6.6	41.9 ± 7.4
			7.0	43.5 ± 8.6	41.1 ± 7.9	38.9 ± 7.6
		3.0	4.0	58.8 ± 2.7	54.4 ± 2.1	52.0 ± 3.8
			5.5	56.2 ± 3.4	50.4 ± 3.0	47.6 ± 3.9
			7.0	55.4 ± 4.8	47.5 ± 5.7	47.4 ± 4.3

M: insect meal, HT: control hydrolysate, HP: Hydrolysate whose insect flour has been pretreated at high hydrostatic pressures. Values represent mean observations of three replicates ± standard deviation. The interaction between treatment, concentration, pH and time was not significant.

**Figure A1:**
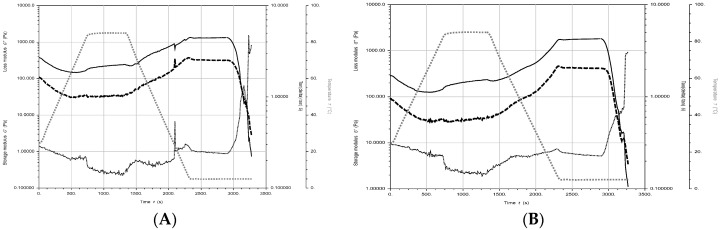
Dynamic moduli G’ and G” of cricket meals at 30% (*w*/*v*) and pH 7.0 during heating and cooling. (**A**) without NaCl addition and (**B**) with 1 M NaCl.

**Figure A2:**
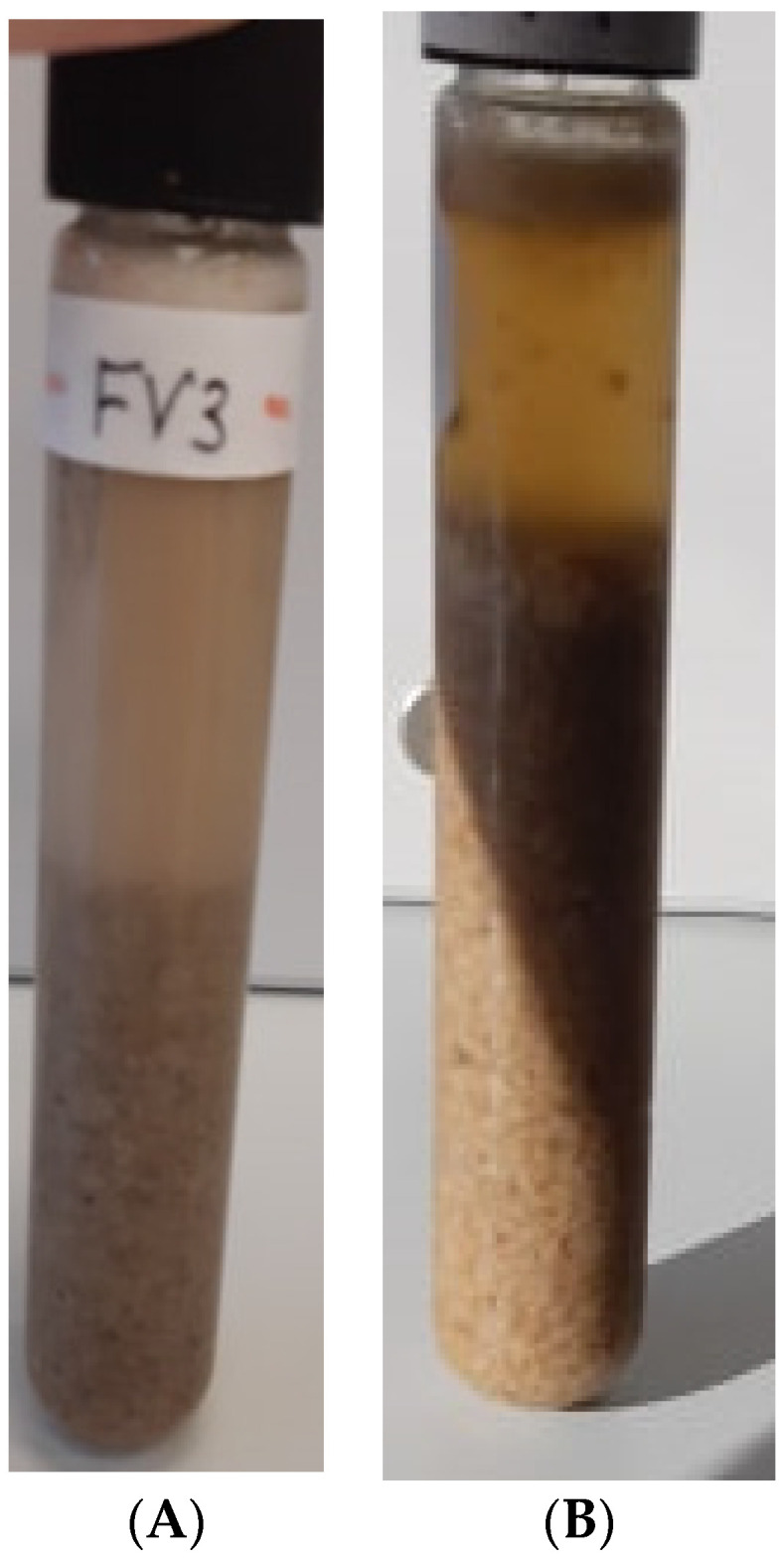
Mealworm meal at 3% (*w*/*v*) and pH 7.0 (**A**) before and (**B**) after heat treatment.

## Appendix A

**Table A1:** Probability values for the effects of insects (crickets and mealworms), treatments (meals, control hydrolysate and hydrolysate generated after HHP treatment of meals), concentration (0.5, 1.0 and 3.0% *w*/*v*), pH (4.0, 5.5 and 7.0) and time (30, 60 and 90 min) on the emulsion stability index.

	Probability Values
Main effects	
Insects (I)	<0.0001
Treatments (T)	<0.0001
Concentration (C)	<0.0001
pH	0.0015
Time	<0.0001
Interactions	
I × T	<0.0001
I × pH	0.9160
T × pH	0.3082
I × C	<0.0001
T × C	<0.0001
C × pH	0.8537
I × Time	0.1246
T × Time	<0.0001
pH × Time	0.7627
C × Time	<0.0001
I × T × pH	0.7393
I × T × C	0.1191
I × C × pH	0.2663
T × C × pH	0.1481
I × T × Time	<0.0001
I × pH × Time	0.1367
I × C × Time	0.4107
T × pH × Time	0.0971
T × C × Time	0.0129
C × pH × Time	0.8910
I × T × C × pH	0.6985
I × T × pH × Time	0.6341
I × T × C × Time	0.4343
I × C × pH × Time	0.8402
T × C × pH × Time	0.7098
I × T × C × pH × Time	0.9184
